# Validation of ligands in macromolecular structures determined by X-ray crystallography

**DOI:** 10.1107/S2059798318002541

**Published:** 2018-03-02

**Authors:** Oliver S. Smart, Vladimír Horský, Swanand Gore, Radka Svobodová Vařeková, Veronika Bendová, Gerard J. Kleywegt, Sameer Velankar

**Affiliations:** a Protein Data Bank in Europe, European Molecular Biology Laboratory, European Bioinformatics Institute, Wellcome Genome Campus, Hinxton, Cambridge CB10 1SD, England; b National Centre for Biomolecular Research, Faculty of Science, Masaryk University, Kamenice 5, 625 00 Brno, Czech Republic; c CEITEC – Central European Institute of Technology, Masaryk University, Kamenice 5, 625 00 Brno, Czech Republic; d Institute of Mathematics and Statistics, Masaryk University, Kotlářská 2, 611 37 Brno, Czech Republic

**Keywords:** PDB, Protein Data Bank, three-dimensional macromolecular structure, validation, ligands

## Abstract

Crystallographic studies of ligands bound to biological macromolecules (proteins and nucleic acids) play a crucial role in structure-guided drug discovery and design, and also provide atomic level insights into the physical chemistry of complex formation between macromolecules and ligands. The quality with which small-molecule ligands have been modelled in Protein Data Bank (PDB) entries has been, and continues to be, a matter of concern for many investigators. Correctly interpreting whether electron density found in a binding site is compatible with the soaked or co-crystallized ligand or represents water or buffer molecules is often far from trivial. The Worldwide PDB validation report (VR) provides a mechanism to highlight any major issues concerning the quality of the data and the model at the time of deposition and annotation, so the depositors can fix issues, resulting in improved data quality. The ligand-validation methods used in the generation of the current VRs are described in detail, including an examination of the metrics to assess both geometry and electron-density fit. It is found that the LLDF score currently used to identify ligand electron-density fit outliers can give misleading results and that better ligand-validation metrics are required.

## Abstract 2

Better metrics are required to be able to assess small-molecule ligands in macromolecular structures in Worldwide Protein Data Bank validation reports. The local ligand density fit (LLDF) score currently used to assess ligand electron-density fit outliers produces a substantial number of false positives and false negatives.

## 1. Introduction   

The quality of small-molecule ligands in Protein Data Bank (PDB) entries has been, and continues to be, a matter of concern for many investigators (Kleywegt & Jones, 1998 ▸; Kleywegt *et al.*, 2003 ▸; Kleywegt, 2007 ▸; Davis *et al.*, 2008 ▸; Liebeschuetz *et al.*, 2012 ▸; Pozharski *et al.*, 2013 ▸; Smart & Bricogne, 2015 ▸; Deller & Rupp, 2015 ▸). Correctly interpreting whether electron density observed in a binding site is compatible with the soaked ligand or represents water or buffer molecules is sometimes far from trivial. It is particularly challenging when ligands are relatively small or bind with only partial occupancy (Pearce *et al.*, 2017 ▸). Low-resolution structures also tend to be more problematic to interpret un­ambiguously, particularly below 3 Å resolution, where any waters mediating interactions between ligand and protein are unlikely to be clearly observed. Furthermore, fitting a ligand into electron density and subsequently refining the model so that it has reasonable stereochemistry, while also fitting the experimental data well, can be challenging, particularly for inexperienced crystallographers (Smart & Bricogne, 2015 ▸). The details of ligand binding are often of crucial importance to the use of a structure, for instance for structure-guided drug discovery (Scapin *et al.*, 2015 ▸). This makes it important to establish dependable metrics that can be used to assess whether a ligand modelled with a macromolecular structure can be relied upon.

The Worldwide PDB (wwPDB) validation report (VR; Gore *et al.*, 2012 ▸, 2017 ▸) provides a mechanism to highlight any major issues concerning the quality of the data or the model at the time of deposition and annotation, so the depositors can fix issues, resulting in improved data quality. In addition, it is useful to help nonspecialist users and referees assess the quality of the coordinate model and supporting experimental data presented in a PDB entry or a manuscript. The first wwPDB/Cambridge Crystallographic Data Centre/Drug Design Data Resource Ligand Validation Workshop (LVW; Adams *et al.*, 2016 ▸) has made recommendations as to how to improve ligand validation.

This paper investigates how the ligand-validation procedures and metrics currently included in the VR work in practice for structures determined by X-ray crystallography.

## 2. Methods   

Analysis of the distribution of ligand-specific metrics reported in the VR was initially performed using the ValTrends^DB^ website (http://ncbr.muni.cz/ValTrendsDB). A current limitation of ValTrends^DB^ is that analysis is performed per PDB entry, with all ligand metric values for that entry being averaged. To get around this limitation, further analysis was performed on an individual ligand basis using *NumPy* (http://www.numpy.org) and *Matplotlib* (https://matplotlib.org/) to plot graphs. The Jupyter Notebook (https://jupyter.org; Shen, 2014 ▸) for the analysis is included in the Supporting Information.

The electron density around selected ligands was visualized using the ligand’s page on the PDBe website (https://pdbe.org) that incorporates the versatile *LiteMol* program (Sehnal *et al.*, 2017 ▸) for interactive three-dimensional visualization of PDB structural models and Electron Density Server (EDS)-style electron-density maps (Kleywegt *et al.*, 2004 ▸) within a web browser.

## 3. Validation of ligand geometric features   

To assess ligand geometry, the wwPDB validation pipeline uses the *Mogul* program (Bruno *et al.*, 2004 ▸) from the Cambridge Crystallographic Data Centre (CCDC). For each bond length and bond angle in the ligand, a search is performed for small-molecule crystal structures in the Cambridge Structural Database (CSD) that have a similar chemical environment.

**Figure 1:**
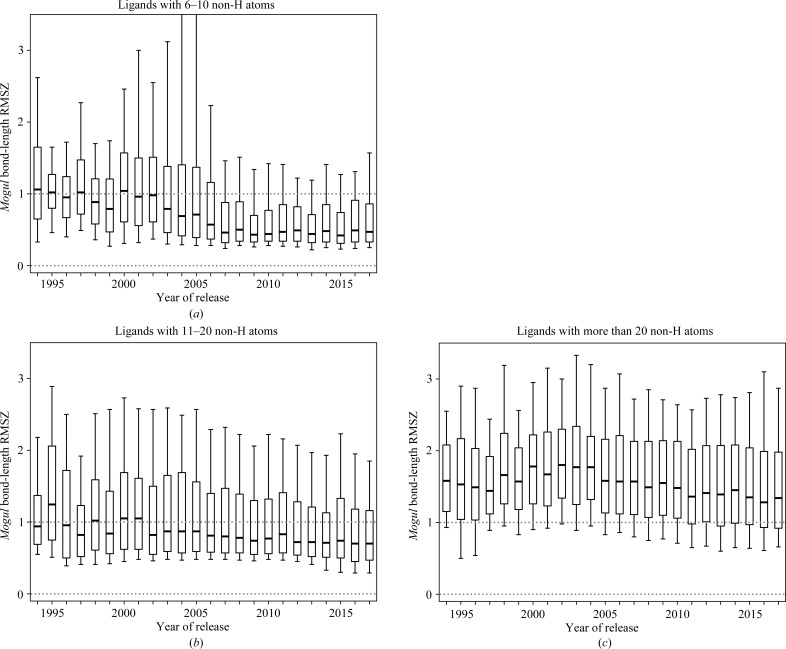
Plots showing how the RMSZ value from *Mogul* analysis of bond lengths for ligands in all PDB depositions solved by X-ray crystallography varies with deposition date and ligand size. The boxes show the upper and lower quartile range, with the thick line marking the median value. The whiskers mark the 10th and 90th percentile of the data, following Kleywegt & Jones (2002 ▸).

Part of the *Mogul* search can be thought of as providing for ligands a CSD survey equivalent to that performed by Engh & Huber (2001 ▸) for ideal values of bond lengths and angles for standard amino acids in proteins. Currently, the VR reports *Mogul* bond-length and bond-angle deviations in terms of *Z*-scores: this is defined as the difference between an observed value and its expected or average value, divided by the standard deviation of the latter. The *Mogul* root-mean-squared *Z*-scores (RMSZ) for all of the bond lengths and angles are calculated for each ligand to allow overall assessments. Fig. 1 ▸ and Supplementary Fig. S1 show the *Mogul* bond-length and bond-angle RMSZ values for PDB structures released in the past 20 years, separated by ligand size. It can be noted that the bond-length RMSZ values depend on ligand size. For ligands with 6–10 non-H atoms recent depositions have a median bond-length RMSZ below 0.5, whereas larger ligands with more than 20 non-H atoms have a median bond-length RMSZ around 1.5. This does not necessarily imply that large ligands are ‘worse’ than small ones, only that bond restraints are relatively easily satisfied in smaller ligands with typical data resolution and electron density. RMSZ values smaller than one are probably caused by the use of the same molecule crystal structures as a source of restraint information and in *Mogul *validation. It can be noted that novel CSD structures could be expected to have RMSZ values for bonds and angles around one, and that values lower than this are not ‘better’. This is particularly important in the fair treatment of structures refined with ligand restraints derived from high-level quantum-chemical procedures (Moriarty *et al.*, 2009 ▸), using a force field (Bell *et al.*, 2012 ▸; Janowski *et al.*, 2016 ▸) or through the direct use of quantum-chemical methods (Borbulevych *et al.*, 2014 ▸; Smart *et al.*, 2016 ▸) to represent the ligands. We conclude that *Mogul* bond-length and bond-angle RMSZ values are not sufficient as ligand-geometry quality metrics and hence cannot be used to assess whether the quality of ligands in the PDB is improving.

It is useful to analyse *Mogul* bond-length and bond-angle results by listing individual bond lengths and angles whose *Z*-score exceeds a threshold value, as is performed in the VR, where ligand bond lengths and angles with an absolute *Z*-score above 2.0 are flagged as ‘outliers’. This value is consistent with that used by Liebeschuetz *et al.* (2012 ▸), but is much lower than the *Z*-score threshold value of 5.0 recommended by the X-ray VTF (Read *et al.*, 2011 ▸) for protein and nucleic acids and used in the corresponding parts of the VR. Using radically different thresholds results in ligand bond lengths and angles being judged far more strictly than those in proteins and nucleic acids, with the routine reporting of a number of moderate ‘outliers’ for well placed ligands refined with reasonable restraints. It would be useful to use a classification that distinguishes between moderate and severe distortion from the *Mogul* expectation, in a similar way to Ramachandran plot analysis (Ramachandran *et al.*, 1963 ▸), where a classification of ‘favoured’, ‘allowed’ and ‘outliers’ is routinely used (Chen *et al.*, 2010 ▸). The LVW (Adams *et al.*, 2016 ▸) recommends that *Mogul* results be presented in the VR using a coloured two-dimensional stick representation, as developed in *Buster-Report* (Global Phasing Ltd, 2011 ▸), that allows the extent of disagreement to be shown clearly.

A complication in interpreting *Mogul* outliers at present is that it is not possible to assess whether an outlier arose because the restraints used in refining the ligand had target values that were not in accord with *Mogul* assessment or because there was a problem in the ligand fit that caused geometric strain in the model. Inclusion of the ligand-related refinement restraints in the structure deposition, as recommended by the LVW (Adams *et al.*, 2016 ▸), will enable the disambiguation of these factors by using *Mogul* to analyse the restraint target values separately from the model.

Read *et al.* (2011 ▸) note that for protein and nucleic acid structures the analysis of bond-length and bond-angle outliers provides only limited geometric validation information, as normally these parameters are tightly restrained using harmonic restraints to ideal values from Engh & Huber (2001 ▸) (for amino acids) or Parkinson *et al.* (1996 ▸) (for nucleotides). Instead, the most useful geometric validation criteria use structural features that are usually not tightly restrained during refinement such as (combinations of) torsion angles or nonbonded contacts (Kleywegt & Jones, 1995 ▸; Kleywegt, 2000 ▸). The *MolProbity* program (Chen *et al.*, 2010 ▸) provides an analysis of the Ramanchandran plot of main-chain torsion-angle combinations (Ramachandran *et al.*, 1963 ▸), as well as an analysis of allowed side-chain rotamers and all-atom nonbonded short contacts; all three criteria are used in the validation slider plots in the VR and are used in the combined overall quality metric (described in §[Sec sec3]3).

*Mogul* analysis of torsion angles and ring puckers has the potential to provide informative validation information for ligand structures (Liebeschuetz *et al.*, 2012 ▸; Smart & Bricogne, 2015 ▸). The current VR includes *Mogul* torsion-angle and ring-pucker analysis, but at present the outlier identification criterion used is loose and only very distorted groups are ever reported. The *CSD-Mercury* (Macrae *et al.*, 2008 ▸) and *Buster-Report* (Global Phasing Ltd, 2011 ▸) tools show how *Mogul* torsion-angle and ring-pucker analysis can be usefully applied in practice.

A limitation in using CSD-derived information from *Mogul* arises when no or too few related small-molecule crystal structures are identified for a particular geometrical feature (for instance a particular bond angle). When this occurs, no assessment can be made of that feature. The current VR does not include information to show which features have *Mogul* statistics and which do not. Coloured two-dimensional stick diagrams (Adams *et al.*, 2016 ▸) will clearly show this information. When no *Mogul* information is available, comparison to the restraints used in refinement becomes particularly important. Indeed, it would be useful if the deposition could include information as to the source of the information used to define a particular restraint, for instance derived from a particular quantum-chemical procedure, so that this could be included in the VR.

Analysis of ligand–protein contacts provides a further means of geometric validation. Currently, the VR uses the *MolProbity* (Chen *et al.*, 2010 ▸) all-atom clash procedure to identify contact distances between the biomacromolecule and ligand that are unreasonably short once H atoms have been added to both. There is the potential to widen validation to include analysis of whether the different functional groups of the ligand make favourable interactions with the biomacromolecule, such as hydrogen bonds or hydrophobic contacts. The *LIGPLOT* (Wallace *et al.*, 1995 ▸) and *PoseView* (Stierand & Rarey, 2010 ▸) programs provide means to display this information in two-dimensional diagrams. The *IsoStar* program (Bruno *et al.*, 1997 ▸) could potentially be used to assess numerically whether the pose and conformation of a ligand are complementary to the protein binding site. Beshnova *et al.* (2017 ▸) have shown how a semi-empirical force field, based on that used by *AutoDock* (Huey *et al.*, 2007 ▸; Morris *et al.*, 2009 ▸), can be used to detect ‘questionable’ ligands in PDB ligand–protein complex structures.

## 4. Assessing ligand fit to electron density   

In addition to assessing the geometric quality of a ligand modelled in a protein, it is crucial to assess whether the electron density supports the placement (that is the presence, location, orientation and conformation) of the ligand (Kleywegt, 2007 ▸; Davis *et al.*, 2008 ▸; Pozharski *et al.*, 2013 ▸; Smart & Bricogne, 2015 ▸; Adams *et al.*, 2016 ▸). It should be noted that the deposition of X-ray structure-factor data only became mandatory in 2008 (Berman *et al.*, 2013 ▸). Because of this, it is not possible to calculate electron-density maps for the 10 409 X-ray PDB entries that were deposited before 2008 without structure-factor data. In these cases, validation is necessarily limited to geometric criteria.

**Figure 2:**
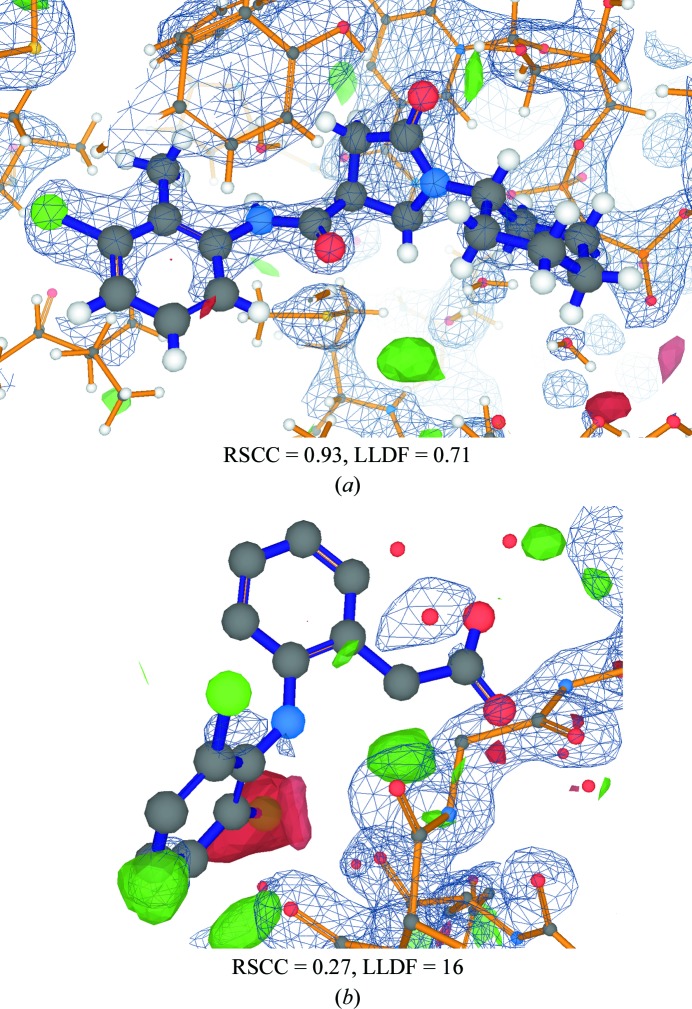
Visualization of two ligands from the PDB together with electron-density maps. The *LiteMol* viewer (Sehnal *et al.*, 2017 ▸) on the PDBe website (Mir *et al.*, 2018 ▸) has been used to visualize the ligands with EDS-style (Kleywegt *et al.*, 2004 ▸) electron-density maps. In each case, the 2*mF*_o_ − *DF*_c_ map is shown as a blue mesh contoured at 0.39 e Å^−3^, whereas the *mF*_o_ − *DF*_c_ difference map is shown by solid green and red surfaces contoured at +0.25 and −0.25 e Å^−3^, respectively. (*a*) Model and electron density for the well placed ligand 468 from PDB entry 4tzt (He *et al.*, 2006 ▸). (*b*) The ligand DIF in PDB entry 3ib0 (Mir *et al.*, 2009 ▸).

Visual inspection of the ligand/protein model together with the electron-density maps provides a powerful way to assess ligand placement (Kleywegt *et al.*, 2004 ▸; Emsley *et al.*, 2010 ▸) and this is particularly important for nonspecialist users of structures. Fig. 2 ▸ shows screenshots of *LiteMol* (Sehnal *et al.*, 2017 ▸) visualizations of ligands in PDB entries and the corresponding EDS-style electron-density maps. Fig. 2 ▸(*a*) shows PDB entry 4tzt (He *et al.*, 2006 ▸), solved at 1.86 Å resolution, where visual inspection confirms that the ligand is well placed in the electron density and that there is little difference density near it. The electron density fully supports ligand placement and conformational details such as ring puckers. In contrast, Fig. 2 ▸(*b*) shows the diclofenac ligand DIF in PDB entry 3ib0 (Mir *et al.*, 2009 ▸) solved at 1.4 Å resolution with an *R*_free_ value of 0.219. The diclofenac ligand modelled in this entry has been classified by Pozharski *et al.* (2013 ▸) as ‘absent’, with the patches of electron density in the region instead being consistent with water molecules (Smart & Bricogne, 2015 ▸). Visual inspection of the ligand together with the electron-density maps is informative and supports this alternative interpretation (Fig. 2 ▸ *b*). The LVW (Adams *et al.*, 2016 ▸) recommends that informative images of the electron-density maps around ligands, as pioneered in *Buster-Report* (Global Phasing Ltd, 2011 ▸), should be provided in the VRs.

Inspection of electron-density maps is useful, but it is highly desirable to have numerical measures to quantify ligand reliability, to enable for instance ranking of ligands in search results and for the selection of sets of reliable protein–ligand complex structures for assessment of the performance of docking programs (Warren *et al.*, 2012 ▸). Currently, the VR provides three numerical metrics to assess how well a ligand fits the EDS 2*mF*_o_ − *DF*_c_ map calculated for that entry.

(i) Real-space *R* value (RSR; Jones *et al.*, 1991 ▸), a measure of how well ‘observed’ and calculated electron densities agree for a ligand. The wwPDB validation pipeline computes RSR using the *MAPMAN* program (Kleywegt *et al.*, 2004 ▸), which compares 2*mF*_o_ − *DF*_c_ and *DF*_c_ maps calculated by *REFMAC*5 (Murshudov *et al.*, 2011 ▸). From a user perspective, it is important to note that the range of RSR is from 0 meaning ‘perfect agreement’, with values approaching or above 0.4 indicating a poor fit and/or low data resolution. A comprehensive description and review of the various approaches to the calculation of RSR values is given by Tickle (2012 ▸).
(ii) Real-space correlation coefficient (RSCC; Jones *et al.*, 1991 ▸). This is an alternative measure of how well the calculated density for a ligand matches the observed electron density. RSCC varies between 1.0 meaning ‘perfect correlation’ and −1.0 meaning ‘perfect anticorrelation’, with values of 0.8 and below indicating a poor fit. The VR User Guide (wwPDB, 2016 ▸) includes a ‘rule of thumb’ for interpreting ligand RSCC based on that used in *Buster-Report* (Global Phasing Ltd, 2011 ▸):
  A value above 0.95 normally indicates a very good fit. RSCC around 0.90 are generally OK. A poor fit results in a value around or below 0.80 that may well indicate the experimental data do not accord with the ligand placement.
(iii) Local ligand density fit (LLDF) compares the RSR value of a ligand with the mean and standard deviation of the RSR values of the neighbouring polymeric standard amino acids and nucleotides. The *CCP*4 (Winn *et al.*, 2011 ▸) program *NCONT* is used to identify standard amino acids and nucleotides that have an atom within 5.0 Å distance of a ligand atom. The mean (〈RSR_site_〉) and standard deviation [σ(RSR_site_)] of the RSR values is then calculated for these neighbouring residues and this is compared with the RSR value of the ligand (RSR_ligand_) to calculate the LLDF score:
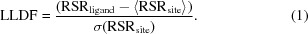
If there are fewer than two neighbouring residues within 5.0 Å of the ligand then the LLDF cannot be calculated.

The LLDF measure was introduced in the VR to provide a measure similar to the normalized RSR values used for polymeric proteins and nucleic acids, RSRZ (Kleywegt *et al.*, 2004 ▸):
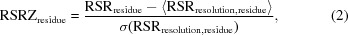
where RSR_resolution,residue_ is the set of RSR values found for that residue type (for example arginine) and resolution range in the PDB. In the VR, amino acids and nucleic acids are reported as electron-density fit outliers if the RSRZ value is above 2.0, and this generally works well.

For most ligand molecules, it would be impossible to calculate RSRZ values as there would be few (and possibly no) occurrences of the ligand type in the PDB. In the absence of any comparable measures in the field, LLDF was introduced as a stop-gap metric where the normalization is against neighbouring standard residues in the binding site. Thus, the ‘normalization’ is carried out internally to a structure, as opposed to externally using many other structures as is the case for RSRZ. In the VRs, all three values (RSR, RSCC and LLDF) are reported. Ligands for which the LLDF value exceeds 2.0 are classified as ‘electron-density fit outliers’ (this value was chosen because it was used as an RSRZ cutoff for standard amino acids). The two examples shown in Fig. 2 ▸ are classified correctly using both the LLDF-based classification and the RSCC ‘rule of thumb’ described above.

However, a number of depositors have reported that the LLDF-based classification marks ligands with reasonable electron-density fit as ‘outliers’ (private communications), and Naschberger *et al.* (2016 ▸) note that LLDF misclassifies reasonably placed solvent molecules such as PEG fragments and glycans. To assess whether these problems are isolated or are more general, an analysis of the LLDF-based classification and the RSCC ‘rule of thumb’ was undertaken for all PDB ligands where the VR includes both values (Table 1 ▸). Table 1 ▸ shows that around a third of PDB ligands are currently classified as ‘outliers’ because they have LLDF values above 2.0. This is a matter of concern, as it indicates either that the PDB ligands are routinely badly placed by crystallographers or that the LLDF-based metric is not reliable. In contrast, just over 11% of the ligands have RSCC lower than 0.8.

**Figure 3:**
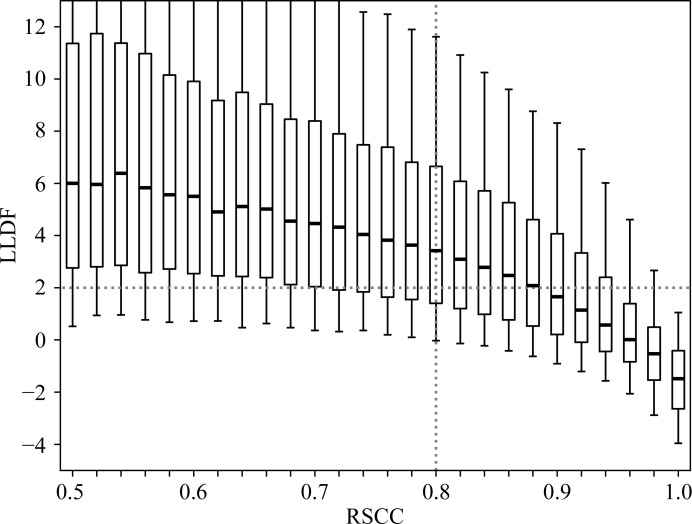
LLDF is plotted *versus* RSCC for all ligands in the PDB for which both values are available (see Table 1 ▸). Box plots and whiskers are as in Fig. 1 ▸.

**Figure 4:**
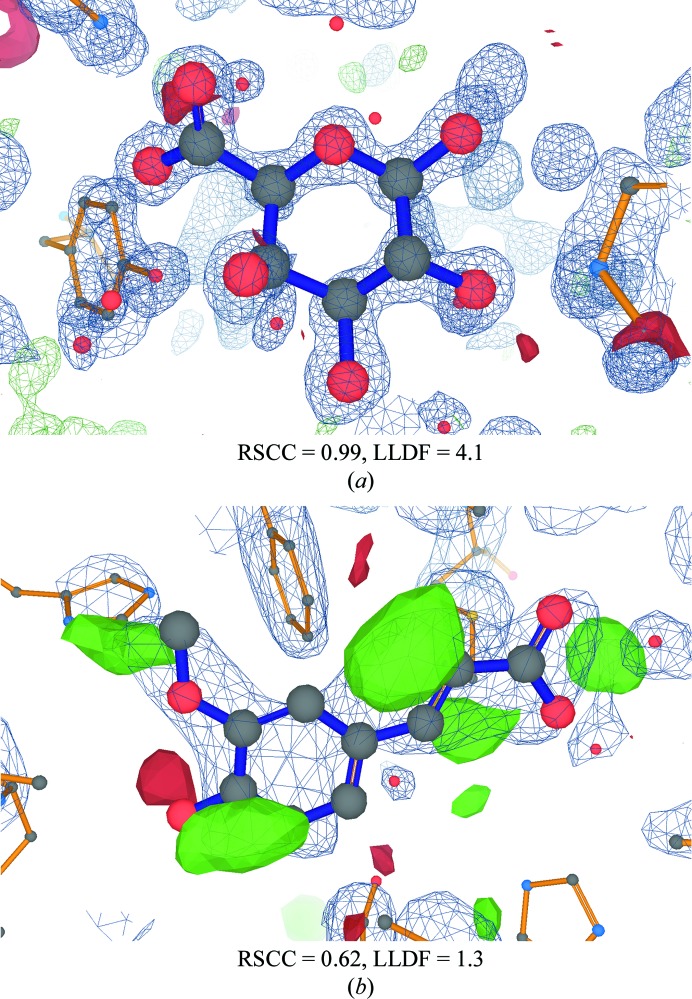
Visualization of two ligands from the PDB together with electron-density maps where LLDF values provide misleading indications. (*a*) Example of a false positive: the map for PDB entry 1kcc at atomic resolution (1.0 Å; Shimizu *et al.*, 2002 ▸) shows electron density for a well placed ligand GTR where each atom is individually resolved. The high RSCC value reflects the good fit. In contrast, the LLDF value is high so that the ligand is incorrectly marked as an electron-density fit outlier in the current VR. (*b*) Example of a false negative: the ligand FER in PDB entry 1kyz (Zubieta *et al.*, 2002 ▸) has a poor fit to the electron density, resulting in large amounts of difference density. The LLDF value of 1.3 results in the ligand not being marked as an electron-density fit outlier in the current VR, whereas the low RSCC value suggests a problem.

Fig. 3 ▸ examines the distribution of LLDF as a function of RSCC. As expected, values of RSCC below 0.8 indicating a poor ligand fit typically correspond to high values of LLDF. As RSCC increases, LLDF generally decreases as both measures reflect that the fit generally improves. Although this is the general trend, the box plots show that there is a wide variation in LLDF values for the same RSCC. Considering ligands with a RSCC value above 0.95 (indicating a very good fit by the ‘rule of thumb’), 14% of these have LLDF values above 2.0 (Table 1 ▸) and thus the ligand involved would be marked as an ‘electron-density fit outlier’ in the VR. To investigate the origin of this anomaly, the EDS electron density for a number of these cases was examined. Curiously, they were often found to be ligands from high-resolution structures with excellent electron-density fits. Fig. 4 ▸(*a*) shows an example where the data resolution is 1.0 Å and the electron density consequently shows atomic detail, with individual separated peaks for each atom (for a review, see Rupp, 2010 ▸). The RSCC for the ligand is 0.99, reflecting its excellent electron density, but since the LLDF value for the ligand is over 4 this ligand is nevertheless marked as an ‘electron-density fit outlier’ in the VR. An explanation for cases like these could be that the RSR values for both the ligand and its surrounding residues are all low and similar in value so that (1) involves division by a very small number (the standard deviation of the RSR values of the surrounding residues) and therefore the computed LLDF values, although correct, become high and are therefore misleading.

**Figure 5:**
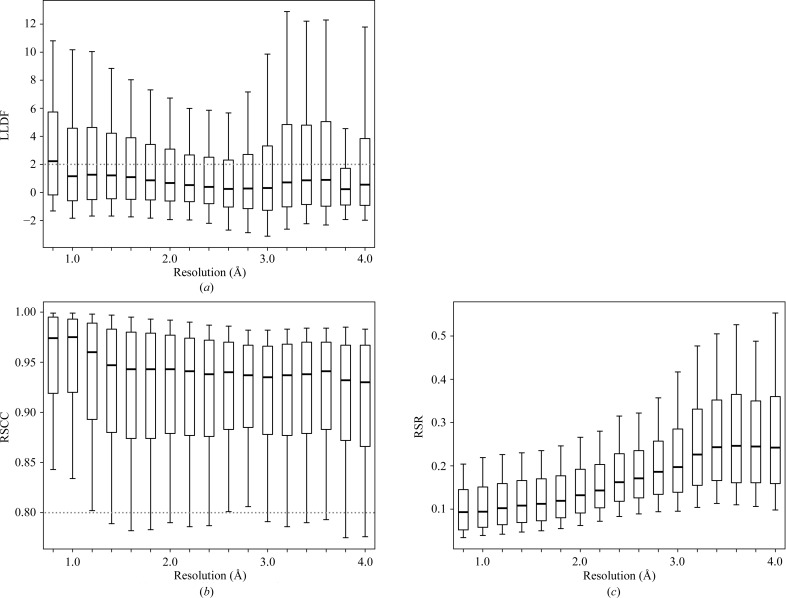
Distribution of (*a*) LLDF, (*b*) RSCC and (*c*) RSR for structures by data resolution for the set of ligands in Table 1 ▸. Box plots and whiskers are as in Fig. 1 ▸.

Further analysis was performed to check whether high LLDF values for reliably placed ligands are a common occurrence for high-resolution structures. Fig. 5 ▸(*a*) shows that the median and upper-quartile LLDF values for ligands in the PDB increase with higher resolutions. Indeed, at 0.8 Å resolution the median LLDF value is around 2, showing that around half the ligands in such high-resolution structures are marked as electron-density fit outliers. The LLDF criterion suggests that structures with a resolution around 2.6 Å have ligands with the fewest ‘electron-density fit outliers’. Although it is possible for ligand placement in high-resolution structures to be problematic, atomic resolution structures are in general the most reliable structures available (Berkholz *et al.*, 2008 ▸; Rupp, 2010 ▸). Hence, LLDF is not a reliable statistic for identifying ligands with poor fit to the electron-density map for high-resolution structures. In contrast, both RSCC and RSR tend to improve for higher resolution structures (Figs. 5 ▸ *b* and 5 ▸ *c*) as ligand electron density becomes more reliable.

A further problem with using LLDF as an ‘outlier’ measure occurs when the electron density of both the ligand and the surrounding residues is poor; an example is shown in Fig. 4 ▸(*b*). This can lead to a low value of LLDF in cases where it would be sensible for the ligand to be marked as an electron-density fit outlier. This phenomenon is caused by the fact that LLDF is an internal measure that expresses how much better or worse the ligand fit is compared with that of its polymeric neighbours. Table 1 ▸ shows that this situation is reasonably common, with around one in four ligands having a poor fit by the RSCC ‘rule of thumb’ being judged as not being outliers by LLDF.

## 5. Discussion and conclusions   

The PDB is a treasure trove of data on the interactions between small-molecule ligands and macromolecules. The assessment of ligand geometry using *Mogul* in the VRs has increased awareness of the issues with ligand geometry, but further work is required to clearly present *Mogul* validation information. The reports also attempt to help with the assessment of electron density for bound molecules and the electron-density model fit quality by providing the LLDF, RSCC and RSR metrics. Our analysis shows that the LLDF metric has drawbacks and is not a reliable metric in several scenarios: (i) for high-resolution structures when all the residues in a binding site have a very good fit to the density and similar numerical values for RSR, (ii) when the electron-density fit for both the ligand and the surrounding residues is poor and (iii) when the ligand has only a small number of surrounding polymeric residues. In such cases, both false positives (good ligands listed as outliers) and false negatives (ligands of questionable quality not identified as outliers) may occur.

There is a clear need for the community to develop better and well tested measures so that they can be incorporated into the wwPDB validation pipeline in the future. The presence of difference electron density close to a ligand may be an indicator of subtle issues of ligand placement such as a chiral inversion or placement of a ring in a reversed orientation (Smart & Bricogne, 2015 ▸). Such difference density can be picked up by visual examination, but metrics that are sensitive to it, such as those provided by *DDQ* (van den Akker & Hol, 1999 ▸), *EDSTATS* (Tickle, 2012 ▸) or *EDIAscorer* (Meyder *et al.*, 2017 ▸), need to be employed (Adams *et al.*, 2016 ▸).

Ligands in low-resolution structures provide particular challenges for validation. As the resolution worsens, the quality of the electron-density map will necessarily deteriorate (Rupp, 2010 ▸). Ligand placement becomes increasingly ambiguous and it is important to take into account geometric considerations: at 3 Å resolution there is no information from the X-ray data to determine the pucker of a ring (Smart & Bricogne, 2015 ▸) and so it is sensible to make sure that the ring is modelled with a geometrically low-strain pucker. Water molecules can be crucial in mediating ligand–protein inter­actions but are unlikely to be observed at resolutions lower than 3 Å, although refinement exploiting information from higher resolution structures can help (Smart *et al.*, 2012 ▸). It is important to note that even if a ligand fits the available electron density well, has good internal geometry and makes sensible interactions with the protein, it does not necessarily mean that the proposed binding mode is correct. How to convey such information to the users of structures in a meaningful and intuitive way is a challenge.

Another issue is how to treat partially ordered ligands where part of the ligand is well defined by the electron density but where another part cannot be defined or unambiguously modelled. At present, depositors take several approaches to modelling the ambiguous part of a ligand: not including the atoms in the model, modelling the atoms but setting the atomic occupancies to zero, or modelling the ligand with normal occupancies and letting the *B* values become very high during refinement. The VR handles these cases by applying normal geometric validation and density-fit validation to all of the atoms that are included in the model. The VR reports the number of zero-occupancy and alternative-conformation atoms in a ligand as well as the number of missing atoms. The LVW (Adams *et al.*, 2016 ▸) has made a recommendation that the PDBx/mmCIF dictionary item _atom_site.calc_f lag be used to identify non-experimentally defined atoms instead of using the atom occupancy. This has advantages, particularly for H atoms included in the model to indicate the modelled charge stage and tautomerization of ligands.

The recent LVW and the second wwPDB X-ray VTF meeting have resulted in recommendations to improve the assessment of ligands and have suggested different metrics to use in the validation reports. These will be implemented in the wwPDB validation pipeline.

## Supplementary Material

Supplementary Figures S1 and S2.. DOI: 10.1107/S2059798318002541/ba5278sup1.pdf

Gzipped Jupyter notebook original with all analysis used in the paper.. DOI: 10.1107/S2059798318002541/ba5278sup2.gz

HTML rendering of the Jupyter notebook with all analysis used in paper (human readable).. DOI: 10.1107/S2059798318002541/ba5278sup3.html

The data for all PDB ligands analyzed.. DOI: 10.1107/S2059798318002541/ba5278sup4.gz

## Figures and Tables

**Table 1:** The fraction of PDB ligands classified as outliers by the LLDF and RSCC ‘rule of thumb’ criteria Analysis of 589 965 ligands in PDB entries, determined by X-ray crystallography and released up to 28 June 2017, where the wwPDB validation report includes values for both LLDF and RSCC. For further details, see the Supporting Information.

	All	RSCC < 0.8 ‘poor’	0.8 < RSCC < 0.95 ‘ok’	0.95 < RSCC ‘very good fit’
All	100%	11.3%	44.9%	43.8%
LLDF > 2 ‘outlier’	34.2%	8.4%	19.8%	6.0%
LLDF < 2 ‘OK’	65.8%	2.9%	25.2%	37.8%
Fraction LLDF ‘outlier’/‘OK’	0.34/0.66	0.74/0.26	0.44/0.56	0.14/0.86
