# Supplementary material for: Validation of ligands in macromolecular structures determined by X-ray crystallography
Source: Acta Crystallogr D Struct Biol. 2018 Mar 2;74(Pt 3):228–36. doi: 10.1107/S2059798318002541 (PMC5947763; doi:10.1107/S2059798318002541)
Supplement: Supplementary file 1 [file d-74-00228-sup1.pdf]

STRUCTURAL
BIOLOGY

Volume 74 (2018)

Supporting information for article:

Validation of ligands in macromolecular structures determined by X-ray crystallography

Oliver S. Smart, Vladimír Horský, Swanand Gore, Radka Svobodová Vareková, Veronika Bendová, Gerard J. Kleywegt and Sameer Velankar

S1. Spearman's Rank Correlation Coefficient

Spearman's Rank Correlation Coefficient ρ provides a useful statistical measure of whether the relationship between two variables is monotonic. ρ takes values from 1 to -1, where 1 indicates perfect association of the rank of variables (so when one increases the other always increases). A value of 0 indicates no association, whereas -1 indicates a perfect negative correlation (when one increases the other always decreases). Table S1 presents ρ for the three ligand-density-fit metrics. The values indicate that there is a moderate negative relationship between RSCC and LLDF. This accords with the observation that metrics normally agree but there are some distinct differences (Table 1 and Figure 3).

Table S1 Spearman's Rank Correlation Coefficients

Analysis is for the set of ligands considered in Table 1.

Metric pair	ρ
RSCC LLDF	-0.56
RSCC RSR	-0.64
LLDF RSR	0.75

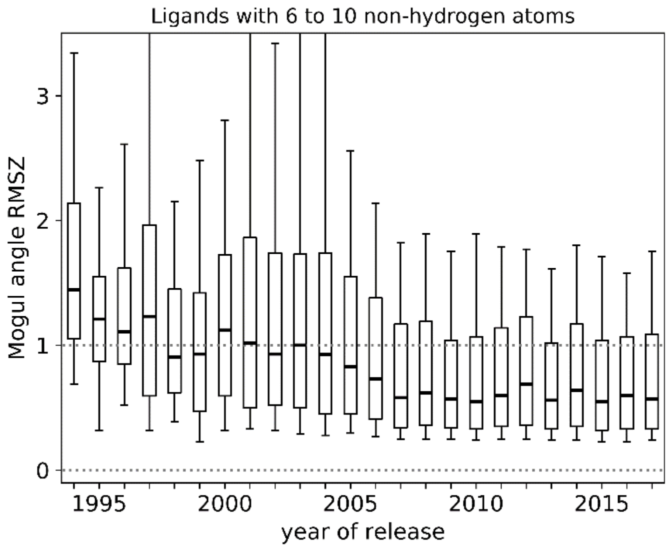
Ligands with 6 to 10 non-hydrogen atoms
Mogul angle RMSZ
0
1
2
3
1995
2000
2005
2010
2015
year of release

(a)

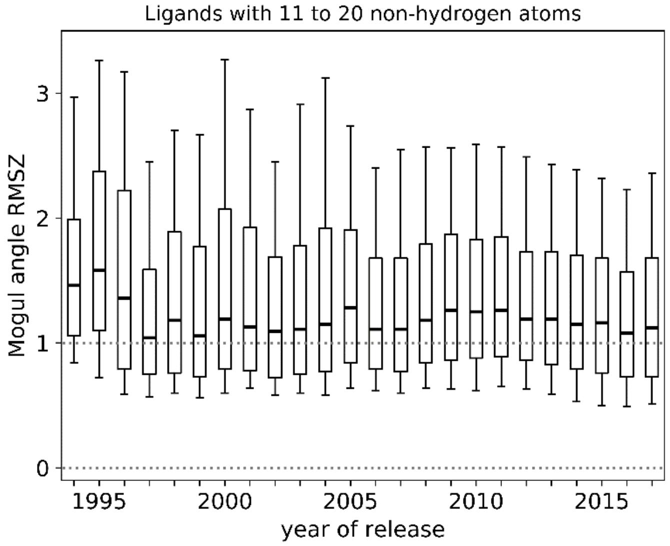
Ligands with 11 to 20 non-hydrogen atoms
Mogul angle RMSZ
0
1
2
3
1995
2000
2005
2010
2015
year of release

(b)

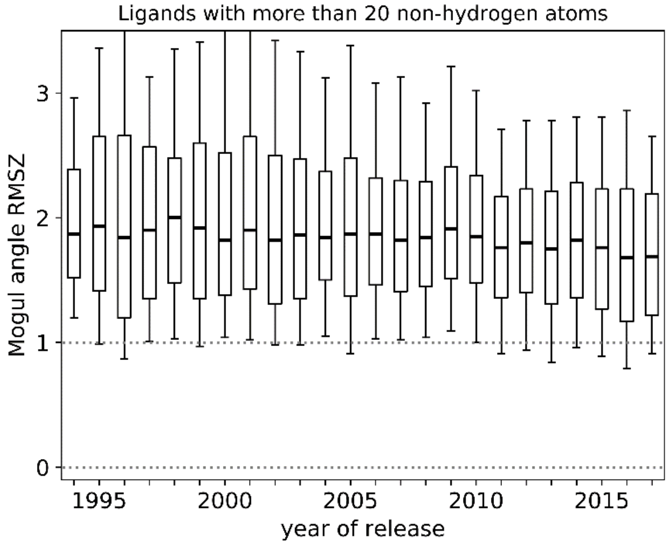
Ligands with more than 20 non-hydrogen atoms
Mogul angle RMSZ
0
1
2
3
1995
2000
2005
2010
2015
year of release

(c)

Figure S1 Plots showing how the RMSZ value from Mogul analysis of bond angles for ligands in all PDB depositions, solved by X-ray crystallography, varies with deposition date and ligand size. The box shows the upper and lower quartile range with the thick line marking the median value. The whiskers mark the 10th and 90th percentile of the data, following Kleywegt and Jones (2002).

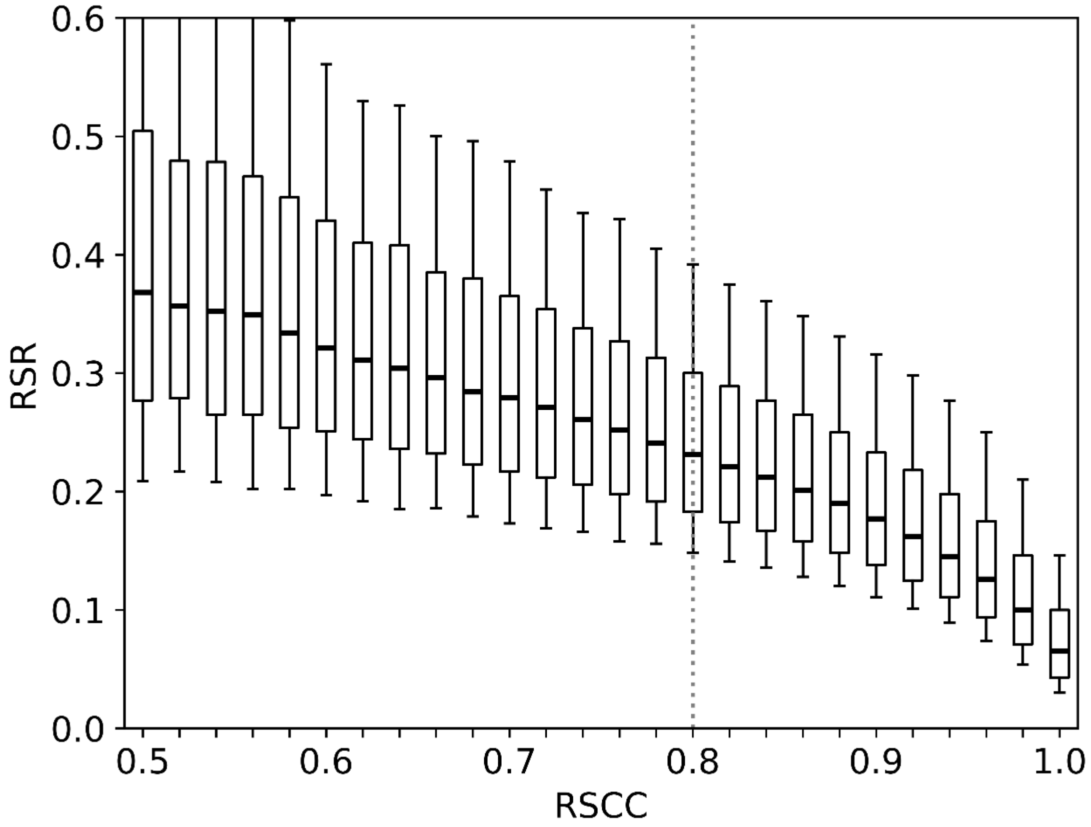

Figure S2 The RSR for ligands is plotted *versus* RSCC for all ligands with LLDF in the PDB (see Table 1). Box plots and whiskers meanings are the same as in Figure S1. It can be seen that ligands with a very good fit by the RSCC “rule of thumb” have low values of RSR with medians approaching 0.1. Poor RSCC is associated with higher RSR but it can be noted that the spread is large.
