# Supplementary material for: Validation of ligands in macromolecular structures determined by X-ray crystallography
Source: Acta Crystallogr D Struct Biol. 2018 Mar 2;74(Pt 3):228–36. doi: 10.1107/S2059798318002541 (PMC5947763; doi:10.1107/S2059798318002541)
Supplement: Supplementary file 3 [file d-74-00228-sup3.html]

ligand\_metric\_analysis\_jupyter\_notebook


# Analysis of ligand metrics from current wwPDB validation reports¶

- Supplementary Materials S2 for *Ligand validation in macromolecular structures determined by Xray crystallography*, Oliver S. Smart, Vladimír Horský, Swanand Gorea, Radka Svobodová Vařeková, Veronika Bendová, Gerard J. Kleywegt and Sameer Velankar, Submitted to Acta D.
- This analysis done in Jupyter Notebook http://jupyter.org/
- using NumPy http://www.numpy.org/
- and Matplotlib https://matplotlib.org/

In [1]:

```
import numpy as np
import numpy.ma as ma
%matplotlib nbagg
import matplotlib as mpl
import matplotlib.pyplot as plt
```

# Ligand data¶

- the ligand data is in a csv file that accompanies this notebook
- this has a a row for each ligand in released PDB entries, determined by X-ray diffraction, up to 28 June 2017
- the columns are labelled:

ID,RELEASE\_DATE,REVISION\_DATE,RESOLUTION,RESNAME,NUMBER\_ATOMS\_NH,N\_ATOMS\_EDS,
RSR,RSCC,LIG\_RSRZ,MOGUL\_BONDS\_RMSZ,MOGUL\_RMSZ\_NUMBONDS,MOGUL\_ANGLES\_RMSZ,MOGUL\_RMSZ\_NUMANGLES

- the data is from wwPDB validation reports (stored in a database).

In [2]:

```
cvs_file = 'ligand_stats_dump.csv'
```

# Analysis of Mogul outliers vs year and ligand size¶

In [3]:

```
# select just the data we want to read
data = np.genfromtxt(cvs_file, dtype=None, delimiter=',', names=True,
                    usecols=('RELEASE_DATE',
                             'NUMBER_ATOMS_NH',
                             'MOGUL_BONDS_RMSZ',
                             'MOGUL_RMSZ_NUMBONDS',
                             'MOGUL_ANGLES_RMSZ',
                             'MOGUL_RMSZ_NUMANGLES'
                            ))
```

In [4]:

```
plot_number = 0
def next_plot_number():
    # method to increment internal plot number for the different graphs
    global plot_number
    plot_number += 1
    return plot_number
```

In [5]:

```
def mogul_vs_year_box_plot(size=None, bond=True):
    """
    plots a box plot Mogul rmsZ bond or angle vs year
    Args:
            size (str): one of 's', 'm' or 'l'
            bond (bool): True means bond, False means angle
    """
    if bond:
        column_mogul_rmsz_num = 'MOGUL_RMSZ_NUMBONDS'
        column_data = 'MOGUL_BONDS_RMSZ'
        ylabel = 'Mogul bond length RMSZ'
        png_out = 'figure_1_bond'
    else:
        column_mogul_rmsz_num = 'MOGUL_RMSZ_NUMANGLES'
        column_data = 'MOGUL_ANGLES_RMSZ'
        ylabel = 'Mogul angle RMSZ'
        png_out = 'figure_s1_angle'
    if size == 's':
        title='Ligands with 6 to 10 non-hydrogen atoms'
        size_select = np.logical_and( data['NUMBER_ATOMS_NH'] > 5, data['NUMBER_ATOMS_NH'] < 11)
        png_out += '_a_small.png'
    elif size == 'm':
        title='Ligands with 11 to 20 non-hydrogen atoms'
        size_select = np.logical_and( data['NUMBER_ATOMS_NH'] > 10, data['NUMBER_ATOMS_NH'] < 21)
        png_out += '_b_medium.png'
    elif size == 'l':
        title='Ligands with more than 20 non-hydrogen atoms'
        size_select = data['NUMBER_ATOMS_NH'] > 20
        png_out += '_c_large.png'
    else:
        raise RuntimeError('unrecognized size')
    enough_data =  data[column_mogul_rmsz_num] > 0
    selection = np.logical_and(size_select, enough_data)
    selected_data = data[selection]
    what_to_plot = column_data
    data_by_year = []
    xaxis_labels = []
    for year in range(1994, 2018):
        releases =  selected_data[selected_data['RELEASE_DATE'] == year]
        this_years_data = releases[what_to_plot]
        data_by_year.append(this_years_data)
        if year%5 == 0:
            xaxis_labels.append(str(year))
        else:
            xaxis_labels.append('')            
    # bigger axis labels 
    font_size = 13
    mpl.rcParams['xtick.labelsize'] = font_size
    mpl.rcParams['ytick.labelsize'] = font_size
    mpl.rcParams['axes.labelsize'] = font_size
    fig = plt.figure(next_plot_number())
    plt.title(title)
    # Create an axes instance
    ax = fig.add_subplot(111)
    # marker for mean https://matplotlib.org/examples/statistics/boxplot_demo.html
    meanpointprops = dict(marker='o', markeredgecolor='black', markerfacecolor='red')
    medianprops = dict(linestyle='-.', linewidth=2.0, color='black')
    nbox = len(data_by_year)
    # Create the boxplot
    bp = ax.boxplot(data_by_year, meanprops=meanpointprops, medianprops=medianprops, 
                    showfliers=False, whis=[10, 90])
    ax.set_xticklabels(xaxis_labels)
    axes = plt.gca()
    ax.yaxis.set_ticks(np.arange(0., 1.8, 0.2))
    ax.set_ylabel(ylabel)
    ax.set_ylim([-0.1,3.5])
    ax.set_yticks([0.,1.0,2.0,3.0])
    # dotted line at 1.0
    ax.plot([0,nbox+1],[1,1], ':', color='gray')
    ax.plot([0,nbox+1],[0,0], ':', color='gray')
    ax.set_xlabel('year of release')
    fig.savefig(png_out, dpi=800, bbox_inches = 'tight', pad_inches = 0.05)
```

In [6]:

```
# first bond graphs for figure 1.
bond=True
for s in ('s', 'm', 'l'):
    mogul_vs_year_box_plot( size=s, bond=bond)
```

In [7]:

```
# Angle graphs for figure S1.
bond = False
for s in ('s', 'm', 'l'):
    mogul_vs_year_box_plot( size=s, bond=bond)
```

# Electron Density Fit.¶

## Re-Read Ligand data to look at Electron Density Fit.¶

In [8]:

```
# select just the data we want to read
data = np.genfromtxt(cvs_file, dtype=None, delimiter=',', names=True,
                    usecols=('RSCC',
                             'LIG_RSRZ',
                             'RESOLUTION',
                             'RSR'
                            )) 
print('Have read data for {} individual PDB ligands'.format(data.shape[0]))
```

```
Have read data for 788371 individual PDB ligands
```

Have data for ~788 000 ligands.

The column `LIG_RSRZ` is actually `LLDF` so rename it:

In [9]:

```
data.dtype.names = ('RSCC', 'LLDF', 'RESOLUTION', 'RSR')
data.dtype.names
```

Out[9]:

```
('RSCC', 'LLDF', 'RESOLUTION', 'RSR')
```

# graphs/analysis of RSCC, RSR and LLDF for ligand¶

### first check small part of the data¶

In [10]:

```
data[0:20]
```

Out[10]:

```
array([(0.992, -5.55, 1.93, 0.033), (0.859, 0.22, 1.93, 0.164),
       (0.79, 0.53, 1.93, 0.23), (0.768, 3.04, 1.93, 0.302),
       (0.859, 3.1, 1.93, 0.235), (0.706, 4.42, 1.93, 0.299),
       (0.647, 4.48, 1.93, 0.291), (0.799, 3.34, 1.93, 0.219),
       (0.679, 12.95, 1.93, 0.279), (0.754, 6.04, 1.93, 0.458),
       (0.918, -2.1, 3.29, 0.147), (0.84, nan, 3.29, 0.214),
       (0.89, nan, 3.29, 0.215), (0.696, 0.17, 3.29, 0.307),
       (0.907, nan, 3.29, 0.193), (0.594, nan, 3.29, 0.254),
       (0.632, nan, 3.29, 0.239), (0.569, nan, 3.29, 0.481),
       (0.668, nan, 3.29, 0.308), (0.918, -1.63, 3.29, 0.18)], 
      dtype=[('RSCC', '<f8'), ('LLDF', '<f8'), ('RESOLUTION', '<f8'), ('RSR', '<f8')])
```

In [11]:

```
# take out nan data
valid_rscc = np.isfinite(data['RSCC'])
valid_rsr = np.isfinite(data['RSR'])
valid_lldf = np.isfinite(data['LLDF'])
valid = np.logical_and(valid_rscc, valid_lldf,valid_rsr)
data_valid = data[valid]
data_valid[0:20]
```

Out[11]:

```
array([(0.992, -5.55, 1.93, 0.033), (0.859, 0.22, 1.93, 0.164),
       (0.79, 0.53, 1.93, 0.23), (0.768, 3.04, 1.93, 0.302),
       (0.859, 3.1, 1.93, 0.235), (0.706, 4.42, 1.93, 0.299),
       (0.647, 4.48, 1.93, 0.291), (0.799, 3.34, 1.93, 0.219),
       (0.679, 12.95, 1.93, 0.279), (0.754, 6.04, 1.93, 0.458),
       (0.918, -2.1, 3.29, 0.147), (0.696, 0.17, 3.29, 0.307),
       (0.918, -1.63, 3.29, 0.18), (0.725, 2.08, 3.29, 0.592),
       (0.904, -1.61, 3.29, 0.179), (0.759, -0.09, 3.29, 0.311),
       (0.914, -1.42, 3.29, 0.174), (0.834, 0.57, 3.29, 0.378),
       (0.904, -2.27, 3.29, 0.139), (0.887, 1.75, 3.29, 0.519)], 
      dtype=[('RSCC', '<f8'), ('LLDF', '<f8'), ('RESOLUTION', '<f8'), ('RSR', '<f8')])
```

In [12]:

```
num_data_valid = data_valid.shape[0]
print('after taking out all nan have data for {} ligands '.format(num_data_valid))
```

```
after taking out all nan have data for 589965 ligands
```

# number of ligands with RSCC, RSR, LLDF data¶

In [13]:

```
num_tot = data.shape[0]
num_valid_rscc = data[valid_rscc].shape[0]
num_valid_rsr = data[valid_rsr].shape[0]
num_valid_lldf = data[valid_lldf].shape[0]

print('number of ligands with a RSCC value is {} that is {:.1f}% of total'.
      format(num_valid_rscc, 100.*num_valid_rscc/num_tot))
print('number of ligands with a RSR  value is {} that is {:.1f}% of total'.
      format(num_valid_rsr, 100.*num_valid_rsr/num_tot))
print('number of ligands with a LLDF value is {} that is {:.1f}% of total'.
      format(num_valid_lldf, 100.*num_valid_lldf/num_tot))
```

```
number of ligands with a RSCC value is 736768 that is 93.5% of total
number of ligands with a RSR  value is 589965 that is 74.8% of total
number of ligands with a LLDF value is 589965 that is 74.8% of total
```

# Table 1 proportion of ligands with LLDF>2 and/or RSCC < 0.8¶

In [14]:

```
print('N.B. analysis is for ligands with LLDF, RSR and RSCC values that are not nan')
lldf_above2 = data_valid[data_valid['LLDF']>=2.0]
num_lldf_above2 = lldf_above2.shape[0]
lldf_below2 = data_valid[data_valid['LLDF']<2.0]
num_lldf_below2 = lldf_below2.shape[0]

rscc_above_0p95 = data_valid[data_valid['RSCC']>0.95]
num_rscc_above_0p95 = rscc_above_0p95.shape[0]
rscc_below_0p8 = data_valid[data_valid['RSCC']<=0.8]
num_rscc_below_0p8 = rscc_below_0p8.shape[0]
rscc_in_between = data_valid[(data_valid['RSCC']<=0.95) & (data_valid['RSCC']>0.8)]
num_rscc_in_between = rscc_in_between.shape[0]
print('number with LLDF>=2 is {} that is {:.1f}% of ligands with values'.
      format(num_lldf_above2, 100.*num_lldf_above2/num_data_valid))
print('number with LLDF<2 is {} that is {:.1f}% of ligands with values'.
      format(num_lldf_below2, 100.*num_lldf_below2/num_data_valid))
print()
print('number with RSCC<=0.8 is {} that is {:.1f}% of ligands with values'.
      format(num_rscc_below_0p8, 100.*num_rscc_below_0p8/num_data_valid))
print('number with RSCC in range 0.8 to 0.95 is {} that is {:.1f}% of ligands with values'.
      format(num_rscc_in_between, 100.*num_rscc_in_between/num_data_valid))
print('number with RSCC>0.95 is {} that is {:.1f}% of ligands with values'.
      format(num_rscc_above_0p95, 100.*num_rscc_above_0p95/num_data_valid))
```

```
N.B. analysis is for ligands with LLDF, RSR and RSCC values that are not nan
number with LLDF>=2 is 201504 that is 34.2% of ligands with values
number with LLDF<2 is 388461 that is 65.8% of ligands with values

number with RSCC<=0.8 is 66391 that is 11.3% of ligands with values
number with RSCC in range 0.8 to 0.95 is 265176 that is 44.9% of ligands with values
number with RSCC>0.95 is 258398 that is 43.8% of ligands with values
```

now look at LLDF values in the different ranges:

In [15]:

```
from collections import OrderedDict
rssc_ranges = OrderedDict()
rssc_ranges[' <=0.8'] = rscc_below_0p8
rssc_ranges['in range 0.8 to 0.95'] = rscc_in_between
rssc_ranges[' >0.95'] = rscc_above_0p95
for key, this_data in rssc_ranges.items():
    print('\nRSCC {}:'.format(key))
    this_lldf_above2 = this_data[this_data['LLDF']>=2.0] 
    num_this_lldf_above2 = this_lldf_above2.shape[0]
    this_lldf_below2 = this_data[this_data['LLDF']<2.0] 
    num_this_lldf_below2 = this_lldf_below2.shape[0]
    print('\t number with LLDF>=2 is {} that is {:.1f}% of ligands with values'.
          format(num_this_lldf_above2, 100.*num_this_lldf_above2/num_data_valid))    
    print('\t number with LLDF<2  is {} that is {:.1f}% of ligands with values'.
          format(num_this_lldf_below2, 100.*num_this_lldf_below2/num_data_valid))
```

```
RSCC  <=0.8:
	 number with LLDF>=2 is 49383 that is 8.4% of ligands with values
	 number with LLDF<2  is 17008 that is 2.9% of ligands with values

RSCC in range 0.8 to 0.95:
	 number with LLDF>=2 is 116619 that is 19.8% of ligands with values
	 number with LLDF<2  is 148557 that is 25.2% of ligands with values

RSCC  >0.95:
	 number with LLDF>=2 is 35502 that is 6.0% of ligands with values
	 number with LLDF<2  is 222896 that is 37.8% of ligands with values
```

# methods to bin data and draw graphs¶

In [16]:

```
def bin_data( this_data, x_col, y_col, bin_increment, bin_low, echo=False, skip=0):
    """
    bin the data into bins and work out the xaxis labels for the graph
    """
    data_in_bin = []
    xaxis_labels = []
    for ic in range(bin_low.size):
        low = bin_low[ic]
        high = low + bin_increment
        select_low = np.greater(this_data[x_col], low)
        select_high = np.less_equal(this_data[x_col], high)
        in_bin = np.logical_and(select_low,select_high)
        this_bin_data = this_data[in_bin]
        this_bin_y = this_bin_data[y_col]
        if ic%5 == skip:
            this_label = '{:.1f}'.format(0.5*(low+high)) 
        else:
            this_label = ''
        if echo:
            print('{:.2f} to {:.2f} label="{}" size={} lower_quartile={:.2f} media={:.2f} upper_quartile={:.2f}'.
                  format( low, high, this_label, this_bin_y.size,
                  np.percentile(this_bin_y,25), np.median(this_bin_y),  np.percentile(this_bin_y,75)))
        data_in_bin.append(this_bin_y)
        xaxis_labels.append(this_label)
    return xaxis_labels, data_in_bin
```

In [17]:

```
def my_plot( fig, x_col, y_col, data_in_bin):
    """common bit of all boxplotes here - supplied with a plt.figure returns the ax for additional bits"""
    # all plot bigger axis labels 
    font_size = 13
    mpl.rcParams['xtick.labelsize'] = font_size
    mpl.rcParams['ytick.labelsize'] = font_size
    mpl.rcParams['axes.labelsize'] = font_size
    # Create an axes instance
    ax = fig.add_subplot(111)
    # marker for mean https://matplotlib.org/examples/statistics/boxplot_demo.html
    # meanpointprops = dict(marker='o', markeredgecolor='black', markerfacecolor='red')
    medianprops = dict(linestyle='-.', linewidth=2.0, color='black')
    nbox = len(data_in_bin)
    # Create the boxplot
    bp = ax.boxplot(data_in_bin, medianprops=medianprops, showfliers=False, whis=[10, 90])
    ax.set_xticklabels(xaxis_labels)
    axes = plt.gca()
    # bigger axis labels 
    font_size = 13
    mpl.rcParams['xtick.labelsize'] = font_size
    mpl.rcParams['ytick.labelsize'] = font_size
    mpl.rcParams['axes.labelsize'] = font_size
    ax.set_xlabel(x_col)
    ax.set_ylabel(y_col)
    return ax
```

# plot LLDF vs RSCC - Figure 3¶

In [18]:

```
x_col = 'RSCC'
y_col = 'LLDF'
# want to bin data in RSCC range from 0.5 to 1.0 
bin_increment = 0.02
bin_low = np.arange(0.49, 1.0, bin_increment)
xaxis_labels, data_in_bin =  bin_data( data_valid, x_col, y_col, bin_increment, bin_low)
fig = plt.figure(next_plot_number())
ax = my_plot(fig, x_col, y_col, data_in_bin)
ax.set_ylim([-5,13]) # LLDF
ax.plot([0,bin_low.size+1],[2,2], ':', color='gray') # LLDF 2.0 dashed line
ax.plot([16,16],[-10,20], ':', color='gray') # RSCC 0.8 line
fig.savefig('figure_3_lldf_vs_rscc.png', dpi=800, bbox_inches = 'tight', pad_inches = 0.05)
```

# LLDF vs Resolution - Figure 5 (a)¶

In [19]:

```
x_col = 'RESOLUTION'
y_col = 'LLDF'
# Resolution bins from 1 to 4
bin_increment = 0.2
bin_low = np.arange(0.7, 4.0, bin_increment)
xaxis_labels, data_in_bin =  bin_data( data_valid, x_col, y_col, bin_increment, bin_low, echo=True, skip=1)
fig = plt.figure(next_plot_number())
ax = my_plot(fig, x_col, y_col, data_in_bin)
ax.plot([0,bin_low.size+1],[2.0,2.0], ':', color='gray') # LLDF 2.0 dotted line
ax.set_xlabel('Resolution in Ångström')
fig.savefig('figure_5a_lldf_vs_resolution.png', dpi=800, bbox_inches = 'tight', pad_inches = 0.05)
```

```
0.70 to 0.90 label="" size=692 lower_quartile=-0.17 media=2.23 upper_quartile=5.73
0.90 to 1.10 label="1.0" size=4303 lower_quartile=-0.59 media=1.15 upper_quartile=4.58
1.10 to 1.30 label="" size=12191 lower_quartile=-0.51 media=1.26 upper_quartile=4.63
1.30 to 1.50 label="" size=26208 lower_quartile=-0.45 media=1.21 upper_quartile=4.22
1.50 to 1.70 label="" size=49169 lower_quartile=-0.49 media=1.09 upper_quartile=3.90
1.70 to 1.90 label="" size=77626 lower_quartile=-0.53 media=0.86 upper_quartile=3.42
1.90 to 2.10 label="2.0" size=91408 lower_quartile=-0.61 media=0.67 upper_quartile=3.09
2.10 to 2.30 label="" size=85364 lower_quartile=-0.66 media=0.52 upper_quartile=2.67
2.30 to 2.50 label="" size=51814 lower_quartile=-0.80 media=0.39 upper_quartile=2.51
2.50 to 2.70 label="" size=46721 lower_quartile=-1.04 media=0.25 upper_quartile=2.31
2.70 to 2.90 label="" size=46557 lower_quartile=-1.15 media=0.28 upper_quartile=2.71
2.90 to 3.10 label="3.0" size=37826 lower_quartile=-1.27 media=0.31 upper_quartile=3.32
3.10 to 3.30 label="" size=41741 lower_quartile=-1.03 media=0.71 upper_quartile=4.84
3.30 to 3.50 label="" size=18306 lower_quartile=-0.86 media=0.86 upper_quartile=4.80
3.50 to 3.70 label="" size=14129 lower_quartile=-0.98 media=0.89 upper_quartile=5.05
3.70 to 3.90 label="" size=2662 lower_quartile=-0.90 media=0.23 upper_quartile=1.72
3.90 to 4.10 label="4.0" size=3153 lower_quartile=-0.92 media=0.55 upper_quartile=3.84
```

# Plot RSCC vs Resolution - Figure 5(b)¶

In [20]:

```
x_col = 'RESOLUTION'
y_col = 'RSCC'
# Resolution bins from 1 to 4
bin_increment = 0.2
bin_low = np.arange(0.7, 4.0, bin_increment)
```

In [21]:

```
xaxis_labels, data_in_bin =  bin_data( data_valid, x_col, y_col, bin_increment, bin_low, echo=True, skip=1)
fig = plt.figure(next_plot_number())
ax = my_plot(fig, x_col, y_col, data_in_bin)
ax.plot([0,bin_low.size+1],[0.8,0.8], ':', color='gray') # RSSC 0.8 dotted line
ax.set_xlabel('Resolution in Ångström')
fig.savefig('figure_5b_rscc_vs_resolution.png', dpi=800, bbox_inches = 'tight', pad_inches = 0.05)
```

```
0.70 to 0.90 label="" size=692 lower_quartile=0.92 media=0.97 upper_quartile=0.99
0.90 to 1.10 label="1.0" size=4303 lower_quartile=0.92 media=0.97 upper_quartile=0.99
1.10 to 1.30 label="" size=12191 lower_quartile=0.89 media=0.96 upper_quartile=0.99
1.30 to 1.50 label="" size=26208 lower_quartile=0.88 media=0.95 upper_quartile=0.98
1.50 to 1.70 label="" size=49169 lower_quartile=0.87 media=0.94 upper_quartile=0.98
1.70 to 1.90 label="" size=77626 lower_quartile=0.87 media=0.94 upper_quartile=0.98
1.90 to 2.10 label="2.0" size=91408 lower_quartile=0.88 media=0.94 upper_quartile=0.98
2.10 to 2.30 label="" size=85364 lower_quartile=0.88 media=0.94 upper_quartile=0.97
2.30 to 2.50 label="" size=51814 lower_quartile=0.88 media=0.94 upper_quartile=0.97
2.50 to 2.70 label="" size=46721 lower_quartile=0.88 media=0.94 upper_quartile=0.97
2.70 to 2.90 label="" size=46557 lower_quartile=0.89 media=0.94 upper_quartile=0.97
2.90 to 3.10 label="3.0" size=37826 lower_quartile=0.88 media=0.94 upper_quartile=0.97
3.10 to 3.30 label="" size=41741 lower_quartile=0.88 media=0.94 upper_quartile=0.97
3.30 to 3.50 label="" size=18306 lower_quartile=0.88 media=0.94 upper_quartile=0.97
3.50 to 3.70 label="" size=14129 lower_quartile=0.88 media=0.94 upper_quartile=0.97
3.70 to 3.90 label="" size=2662 lower_quartile=0.87 media=0.93 upper_quartile=0.97
3.90 to 4.10 label="4.0" size=3153 lower_quartile=0.87 media=0.93 upper_quartile=0.97
```

# Plot RSR vs Resolution - Figure 5(c)¶

In [22]:

```
x_col = 'RESOLUTION'
y_col = 'RSR'
bin_increment = 0.2
bin_low = np.arange(0.7, 4.0, bin_increment)
xaxis_labels, data_in_bin =  bin_data( data_valid, x_col, y_col, bin_increment, bin_low, echo=False, skip=1)
fig = plt.figure(next_plot_number())
ax = my_plot(fig, x_col, y_col, data_in_bin)
ax.set_xlabel('Resolution in Ångström')
fig.savefig('figure_5c_rsr_vs_resolution.png', dpi=800, bbox_inches = 'tight', pad_inches = 0.05)
```

# Plot RSR vs RSCC - Figure s2¶

In [23]:

```
x_col = 'RSCC'
y_col = 'RSR'
# want to bin data in RSCC range from 0.5 to 1.0 
bin_increment = 0.02
bin_low = np.arange(0.49, 1.0, bin_increment)
xaxis_labels, data_in_bin =  bin_data(  data_valid, x_col, y_col, bin_increment, bin_low)
# Create a figure instance
fig = plt.figure(next_plot_number())
ax = my_plot(fig, x_col, y_col, data_in_bin)
ax.set_ylim([0,0.6]) # RSR
ax.plot([16,16],[-10,20], ':', color='gray') # RSCC 0.8 line
fig.savefig('figure_s2_rsr_vs_rscc.png', dpi=800, bbox_inches = 'tight', pad_inches = 0.05)
```

# Spearman's rank correlation coefficient - Table S1¶

- this is a useful statisical measure whether the relationship between two variables is monotonic.
- For a better explanation see https://en.wikipedia.org/wiki/Spearman%27s\_rank\_correlation\_coefficient
- Thanks to https://www2.warwick.ac.uk/fac/sci/moac/people/students/peter\_cock/python/rank\_correlations/ for explaining how to calculate in practice.

In [24]:

```
from scipy.stats import spearmanr # use scip Spearman implementation
```

In [25]:

```
print("Spearman's rank correlation coefficients:")
for (pair_a, pair_b) in (('RSCC', 'LLDF'), ('RSCC', 'RSR'), ('LLDF', 'RSR')):
    spearman_r = spearmanr(data_valid[pair_a],data_valid[pair_b])
    print('{} {}\tcorrelation={:5.2f}\tp value={}'.format(pair_a, pair_b, spearman_r[0], spearman_r[1]))
```

```
Spearman's rank correlation coefficients:
RSCC LLDF	correlation=-0.56	p value=0.0
RSCC RSR	correlation=-0.64	p value=0.0
LLDF RSR	correlation= 0.75	p value=0.0
```

In [ ]:

```

```
